# The Ever-Growing Cost of Hospital Construction

**Published:** 1899-07-08

**Authors:** 


### The Institutional Workshop.

### THE EVER-GROWING COST OF HOSPITAL CONSTRUCTION.

Whichever way we turn, whether in town or country, we hear of new hospitals being built, or of old ones being converted to modern form. In some places these are cottage hospitals; in others we see large and stately buildings being erected, one may almost say regardless of expense, with the definite aim of putting their inmates in the best possible position for recovery whatever the cost may be; in other towns we see great hospitals being built by boards of guardians, separate infirmaries as they are called, but really general hospitals complete in every particular; and in others, again, fever hospitals are being established by sanitary authorities. But wherever we go we meet with the same phenomenon, namely, a steadily growing expenditure per bed provided; and we cannot but see that not only is the expenditure upon hospitals becoming a yearly growing strain both upon charity and rates, but that this expensiveness of construction is likely to stand much in the way of that extension of the hospital system which seems an almost necessary corollary of recent advances in medical science, and the question is strongly forced in upon us as one which must be asked in all seriousness, Can nothing be done to check this ever-growing cost of hospital construction?

First of all, as to what this increase really is. We may be told that years and years ago the Hôtel Dieu in Paris and St. Thomas's in London cost far more than is being spent on any hospital that is now being put up, but these have always been looked upon as fancy buildings of quite exceptional character and position, and if we turn back to our own experience and our own inquiries in regard to ordinary hospitals erected at about the time referred to we are sure that £250 a bed was an estimate for which what were then looked upon as good and useful hospitals were capable of being erected. But what do we see now?

The following is a list of a few hospitals erected in recent years, giving the number of beds and the cost per bed, without including land or furniture:—

| | | | |
|---|---|---|---|
| Lancaster Infirmary | ... 60 beds, | cost £466 | per bed. |
| Halifax Royal Infirmary ... | 150 ,, | £486 | ,, |
| Lightburn Fever Hospital | 60 ,, | £483 | ,, |
| Croydon Fever Hospital ... | 50 ,, | £451 | ,, |

(This is for permanent buildings, the temporary structures having cost only £55 per bed.)

Park Hospital (Fever) ... 548 beds, cost over £500 per bed.
Grove Hospital (Fever)... 518 beds, cost £431 per bed.
Brook Hospital (Fever)... 488 beds, cost about £500 per bed.

What, then, are the causes which have led to the increased cost of hospitals erected in recent years?

First we must place the increased cost of labour, which tells upon every individual item in the estimate. This is not a matter of masons' and carpenters' wages alone. The wages question affects the price of every article employed, as well as the final process of putting the building together, and the result is that of late years the cost of building has increased at the rate of about 5 per cent. per annum, and sometimes, as when it has happened that workmen have got an additional penny per hour and the cost of materials has risen at the same time, has jumped up 10 per cent. in a single year.

Secondly, we have to consider what may be termed change of fashion in hospital construction, which always seems to have an upward tendency. This is shown by the constantly increasing demand for more space, more expensive materials, more costly modes of construction —such as the pavilion plan, one-storey buildings, placing the wards on arches, &c.—new and more elaborate machinery, costlier sanitation and more expensive sanitary fittings, and vastly better accommodation for the nurses than used to be considered sufficient. This change of fashion is partly an expression of real progress, but one must not be blind to the fact that it is in part experimental and due to evanescent theories and to the inventive efforts of architects and manufacturers who are constantly elaborating new and apparently better ways of doing things. As a third cause of the increased cost of hospitals is the ever-growing increase in the staff which is thought necessary for their proper conduct. This is an item which shows a constant tendency to advance, so much so that the ideal of "one nurse to each patient" seems by no means impossible of attainment. How far we have already got in this matter of staff may be gathered from a report of the Metropolitan Asylums Board, from which it appears that in some of the fever hospitals under their care the proportion of staff to patients is as high as 1 to every 1·4 patients, and of nurses 1 to every 3 patients. How seriously this increases even the initial cost of a hospital, not to mention its upkeep afterwards, may be understood when we consider that bedrooms, dining-rooms, and sitting-rooms have to be provided for all these people, together with baths and sanitary appliances of all kinds, the latter of which have to be re-duplicated over and over again.

We find in modern hospitals house surgeons, a matron and her assistants, sisters, nurses and probationers, servants of all sorts, porters, engineers, laundry women, gatekeepers, coachmen, gardeners, &c. With better nurses has grown a demand for separate bedrooms, a demand which must be met if good nurses are to be obtained. Again, if discipline is to be kept up, the sisters must have a sitting-room distinct from that used by the nurses and probationers. The matron also no longer does her accounts and sees her nurses in her sitting-room, but has to be provided with an office. If good house surgeons are to be obtained each must have his own sitting-room as well as his own bedroom. Even spare bedrooms have to be provided in case illness should make it necessary to obtain the presence of a "locum." A hospital must now have at least three dining-rooms, one for staff, one for nurses, and one for servants, and in modern hospitals there may be for these three messes, three sets of glass and china, and three separate pantries. Then consider the bath-rooms and sanitary appliances, separate sets of which have to be supplied for house surgeons, matron, nurses, servants, and for the engineers and porters, and all this in addition to those which are provided for the patients.

When, then, we find that modern hospitals cost about £500 per bed, we must remember that this is calculated on the number of beds for patients, not on the total number of beds in the institution, which is far greater now for a given number of patients than used to be the case. Not only, then, does each brick that is laid and each bed that is provided cost more than they used to do, but in addition to the patients' beds, on the number of which the cost is calculated, a whole host of other beds have to be supplied, most of which, from being placed in separate rooms and furnished with their proportion of sitting-rooms, &c., are really more expensive than an equivalent number of beds in patients' wards would be.

The fourth and last cause of increased cost to which we would draw attention is the growing desire for architectural ornament. This applies not only to the buildings, but to the gates, fences, gardens, and general surroundings; and although it is often said that, spread over the total, the difference between a handsome and a plain building is not very great, still it is considerable, and there can be no doubt that the taste for ornamentation in buildings, or, at the least, the distaste for hideousness, is a growing one, and all this plays its part in the increasing cost of hospital construction. If, then, we ask where all this is going to end, and how far we are from finality in the matter of cost, we must ask whether or not the causes of the increased cost are or are not likely to go on? (1) He would, indeed, be an optimist who would dare to hope that the cost of building will get less. Everything seems to point to a steady increase in the cost of both labour, material, and supervision. (2) The staff will probably continue for some time yet to grow. In every form of service people tend to do less in a given time than they used to do, and to ask for better accommodation and shorter hours than they used to be contented with; and the more nursing becomes a service, a mode of getting a living, and the more it becomes divorced from that religious enthusiasm which led its earlier devotees to submit to long hours, hard work, and bad conditions as necessary parts of their sacred mission, the more will nurses follow the custom of the day and demand higher wages, shorter hours, and better accommodation. No one, then, who considers the number of hours that some nurses are still on duty can imagine that we have yet reached finality in the matter of staff, although it is just possible that with lessened hours of work the employment of non-resident nurses may some time in the future tend to lessen the amount of nurses' accommodation required in some sorts of hospitals. (3) How far medical men will continue to ask in the name of medical science for more elaborate and more expensive accommodation for their patients it is difficult to say, but it does not seem likely that they will lessen their demands, or that they will any less than in the past continue to be swayed by fashion. In fact, the rapidity with which new ideas on all sorts of medical matters now become popularised makes it all the more difficult for a sober-minded surgeon to hold his hand. When everyone talks as if he knew all about germs and the requirements of aseptic surgery, a surgeon who puts up with a wooden operating table runs the risk of being considered out of date, and it is the same all round. With each new theory new demands are likely to be made and new appliances asked for, not entirely because they are known to be of practical utility, but to a large extent because they accord with the theory of the day, and are obviously essential to logical completeness in hospital construction —and we do not think we have yet attained finality in medical theorising. At the present moment, with the marble floors and glazed walls and resplendent fittings with which our hospital wards are so liberally supplied, one might think that even the doctors would hesitate to ask for more. But we are not sure. It is by no means certain that before another ten years are over we may not find them asking for separate wards with nurses' room attached, in which each operation case with its two nurses may remain isolated from the rest of the community; while probably very shortly will come the request for a completely separate staff for dealing with septic and suspicious cases. So that when we are asked where all this is going to end we can only answer that certainly we have not got to the end yet.

When the burden becomes absolutely intolerable we shall, perhaps, begin to see light. Then, perhaps, some classification will take place both in regard to hospitals and patients, and just as we now send convalescents to institutions where they can be looked after for much less money than they would cost in perfectly-fitted hospitals, so we shall probably weed out many patients who will do quite well in hospitals less expensively arranged than those which are provided with every appliance necessary for the gravest operative work.

Moreover, we are not entirely without hope that with the evolution of a special class of architects skilled in hospital construction some economy may result. As things are done at present it is too often the case that when a new hospital is required a certain number of the committee, having visited other hospitals and educated themselves up to a certain point in hospital construction, employ a selected number of architects to draw up competing plans, architects who know perfectly well that their plans will not be accepted unless they squeeze into them all the little fads and dodges which have appeared in all the hospitals erected within the last dozen years. And so they "paint the lily." What is obvious in looking over many modern hospitals is that a strong architect who knew his business could often, by saying "that is good enough," have saved expense.

